# Investigating microRNAs as biomarkers in disorders of consciousness: a longitudinal multicenter study

**DOI:** 10.1038/s41598-023-45719-7

**Published:** 2023-10-27

**Authors:** Nicolò Musso, Dalida Bivona, Carmelo Bonomo, Paolo Bonacci, Maria Enza D’Ippolito, Cristina Boccagni, Francesca Rubino, Antonio De Tanti, Lucia Francesca Lucca, Valeria Pingue, Valentina Colombo, Anna Estraneo, Stefania Stefani, Maria Andriolo, Sergio Bagnato

**Affiliations:** 1 Department of Biomedical and Biotechnological Sciences, University of Catania, 95123 Catania, Italy; 2 Molecular Biology Laboratory, Giuseppe Giglio Foundation, 90015 Cefalù, Italy; 3 Unit of Neurophysiology and Unit for Severe Acquired Brain Injuries, Giuseppe Giglio Foundation, 90015 Cefalù, Italy; 4 Cardinal Ferrari Center, 43012 Fontanellato, Italy; 5 RAN (Research in Advanced Neuro-Rehabilitation), S. Anna Institute, 80067 Crotone, Italy; 6 Neurorehabilitation and Spinal Units, Istituti Clinici Scientifici Maugeri IRCCS, 27100 Pavia, Italy; 7 Montecatone Rehabilitation Institute, 40026 Imola, Italy; 8 Don Gnocchi Foundation IRCCS, 50124 Florence, Italy; 9 Clinical Pathology Laboratory, Provincial Health Authority of Caltanissetta, 93100 Caltanissetta, Italy

**Keywords:** Neurological disorders, Trauma

## Abstract

MicroRNAs (miRNAs) are involved in gene regulation and may affect secondary brain injury and recovery in patients with disorders of consciousness (DoC). This study investigated the role of five miRNAs (150-5p, 132-3p, 23b-3p, 451a, and 16-5p) in prolonged DoC. miRNA levels were assessed in serum samples from 30 patients with unresponsive wakefulness syndrome or minimally conscious state due to traumatic or hypoxic-ischemic brain injury (TBI, HIBI) at baseline (1–3 months) and 6 months post-injury. Patients’ diagnoses were determined using the Coma Recovery Scale revised, and functional outcomes were evaluated 6 months after injury with the Glasgow Outcome Scale Extended (GOSE) and the Functional Independence Measure (FIM). Compared to healthy controls, patients with TBI had lower levels of miRNAs 150-5p, 132-3p, and 23b-3p at baseline, while patients with HIBI had lower levels of miRNA 150-5p at baseline and 6 months post-injury and a reduction of miRNA 451a at baseline. Higher levels of miRNAs 132-3p and 23b-3p were associated with better outcomes in TBI patients as indicated by GOSE and FIM scores. This study highlights distinct miRNA dysregulated patterns in patients with prolonged DoC, dependent on etiology and post-injury time, and suggests that miRNAs 132-3p and 23b-3p may serve as prognostic biomarkers.

## Introduction

The vegetative state, also known as unresponsive wakefulness syndrome (UWS)^1^, is the worst possible clinical evolution in comatose survivors of severe brain injuries. Patients with UWS exhibit no behavior indicative of self-awareness or awareness of the external world and they can remain unconscious indefinitely or progress to a minimally conscious state (MCS), which is characterized by the fluctuating reappearance of minimal signs of awareness^2^. When UWS or MCS persist for more than 28 days, they are defined as prolonged disorders of consciousness (DoC)^3^. Severe brain injuries, such as traumatic brain injuries (TBI) and hypoxic-ischemic brain injuries (HIBI), can cause long-term structural and functional changes in the brain that may contribute to both neurodegeneration and recovery of brain functions^4–6^; the mechanisms of secondary brain injury and restorative brain plasticity occurring simultaneously in these patients are still largely unknown. As a result, to make an accurate prognosis for recovery of consciousness and functional outcomes is a major challenge for physicians treating patients with prolonged DoC. Current prognostic recommendations suggest a multimodal approach that includes serial standardized behavioral assessments, neurophysiological techniques, and neuroimaging studies^7^. However, the results are not yet satisfactory, and there is a pressing need for new biomarkers to be incorporated into current prognostic models to improve their sensitivity. In this context, circulating microRNAs (miRNAs) are good candidates, since each miRNA can reflect large-scale and long-term genetic reorganizations following acute brain injuries^8^.

miRNAs are a large class of small (18–25 nucleotides) non-coding RNAs that regulate gene expression in both physiological and pathological contexts^8^. miRNAs act by degrading messenger RNAs, suppressing translation, or both, by binding to partially complementary sites in the 3′ untranslated region of target genes^9^. To date, approximately 2000 miRNAs have been identified, which regulate the expression of about 60% of all genes and play a central role in many biological processes such as cell cycle, cell metabolism, apoptosis, and immune response^10,11^. Circulating miRNAs can be detected using digital PCR (dPCR), a highly sensitive technique, and have been proposed as emerging biomarkers for both acute brain injury and neurodegenerative diseases^12,13^. The analysis of miRNAs can provide a powerful tool for understanding the pathophysiology of prolonged DoC and predicting patient outcomes. In particular, the analysis of miRNAs in patients with prolonged DoC can provide valuable insights into the molecular mechanisms occurring during brain reorganization following a severe brain injury.

In this longitudinal multicenter study, we selected five miRNAs involved in acute brain injury or chronic neurodegeneration (miRNAs 16-5p, 150-5p, 132-3p, 23b-3p and 451a) and evaluated them in patients with prolonged DoC caused by TBI or HIBI. Following TBI, a reduction in miRNA 16-5p levels has been observed both in plasma during the acute phase and in the brain of patients with fatal TBI^14,15^. Changes in 150-5p levels have been observed both in traumatic injuries and neurodegenerative diseases^16,17^. miRNA 132-3p and 23b-3p have been identified as regulators of inflammatory and apoptotic pathways implicated in HIBI and neurodegenerative diseases^18–20^. Lastly, miRNA 451a has been recognized for its role in governing apoptotic pathways following ischemia/reperfusion injury^21^.

Our study aimed to provide a better understanding of the long-term genetic reorganization that occurs after a severe brain injury and to identify new potential biomarkers for patients with prolonged DoC.

## Methods

### Participants

This multicenter longitudinal study enrolled patients admitted to five Italian centers specialized in the rehabilitation of patients with prolonged DoC after an acute brain injury. Patients were included if they met the following criteria: (i) diagnosis of UWS or MCS according to the Coma Recovery Scale-Revised (CRS-R) at the time of enrollment, (ii) DoC caused by TBI or HIBI, (iii) enrollment at 1–3 months after brain injury, (iv) clinical and miRNA data availability at baseline and 6 months post-injury, and (v) age ≥ 18 years. Exclusion criteria were (i) previous brain injury and (ii) unstable clinical condition (e.g., hemodynamic instability, severe respiratory failure, or acute hydrocephalus). Patients’ data were compared with those of age-matched healthy controls without previous history of neurological disease who were recruited from blood donors and healthcare personnel at the coordinating center.

The study was conducted according to the guidelines of the Declaration of Helsinki and was approved by the regional ethics review board (Palermo 1 Ethics Committee, Palermo, Italy: verbal no. 10/2021) of the coordinating center (Giuseppe Giglio Foundation, Cefalù, Italy). The protocol was then ratified by the ethics committees of the other centers involved in the study. Patients’ legal guardians and healthy participants provided written informed consent which was collected and stored at each participating center.

### Clinical evaluation

At enrollment, all patients underwent standard neurological examination and assessment with the Italian version of the Coma Recovery Scale Revised (CRS-R)^22^ at least 5 times over 3 consecutive days. The CRS-R is the most reliable tool for behavioral assessment in patients with DoC following a coma and is used to diagnose UWS, MCS, and emergence from MCS^23,24^. It consists of 23 hierarchically organized items organized into six subscales evaluating auditory, visual, motor, oromotor/verbal, communication, and arousal functions. The total score ranges from 0 (coma) to 23 (emergence from MCS). To minimize the risk of misdiagnosis, UWS and MCS diagnoses were accepted only if confirmed at all 5 evaluation timepoints^25^.

Outcomes were assessed 6 months post-injury using the CRS-R, the extended Glasgow Outcome Scale (GOSE) and the Functional Independence Measure (FIM). The GOSE is a 1–8 global scale with the categories of death (score of 1), vegetative state, severe disability (lower and upper), moderate disability (lower and upper), and good recovery (lower and upper; scores of 7 and 8)^26^. The FIM is an 18-item, seven-level ordinal scale that includes measures of independence for self-care, sphincter control, transfers, locomotion, communication, and social cognition^27^.

### Total RNA purification

At the end of initial and follow-up clinical evaluations, blood samples for miRNAs analysis were collected from patients into serum-separating tubes. Samples were centrifuged at 1500 rpm for 15 min and stored at − 80 °C until use. Total RNA was purified using the miRNeasy Serum/Plasma Advanced Kit (cat. No. 217204, QIAGEN) from 200 μL of serum, following the manufacturer’s instructions. This kit maximizes the recovery of small RNAs, including miRNAs, and is optimized for serum samples.

### miRNAs quantification

Capillary electrophoresis is considered the most reliable method for accurately determining the integrity and the quantification of miRNAs, that are the major factors affecting the outcome of downstream expression analyses^28^. Both quantity and quality of miRNAs have been assessed through chip-electrophoresis using the Agilent Small RNA Kit (cat. no. 5067-1548, Agilent Technologies) on the Agilent 2100 Bioanalyzer.

### cDNA synthesis

Starting from a fixed RNA input of 2.5 μL, cDNA synthesis was carried out using the miRCURY LNA™ RT Kit (cat. no. 339340, QIAGEN) according to the manufacturer’s instructions. As recommended, cDNAs were diluted 1:30 prior to dPCR.

### dPCR

Absolute quantification (positive particle = copies/μL) of miRNAs was performed through dPCR with QIAcuity EG PCR Kit (cat. no. 250111, QIAGEN) and 26k-partitions nanoplates (cat. no. 250001, QIAGEN) on QIAcuity One platform. The used primers are listed in Supplementary Table 1. Thermal cycling and imaging were performed following the manufacturer’s instructions.

### Statistical analysis

Demographic and clinical data are presented as medians (interquartile range). The Mann–Whitney *U* test for continuous variables, and the chi-square test for categorical variables were used to compare demographic and clinical data between patients and healthy controls, and between groups of patients (TBI vs. HIBI) as well.

The Student’s *t*-test was used to compare miRNA levels between patients and healthy controls and between miRNA levels at 1–3 months (baseline) and 6 months post-injury.

Correlations among all miRNA expressions were evaluated in healthy controls and patients (at baseline and six-month post-injury) using Pearson’s correlation coefficients in correlation matrixes. Correlation coefficients range from − 1 to + 1, where − 1 indicates a total negative correlation between two miRNAs, 0 no correlation, and + 1 a total positive linear correlation. The R-Squared was also calculated to measure the reliability of the linear relationship among miRNA expressions included in the model. Its value ranges from 0 (fully correlated miRNAs) to 1 (non-correlated miRNAs).

Multiple linear regression was used to determine the effect of each miRNA and was adjusted for the effects of the other miRNAs. A linear model was constructed, in which the observed (dependent) variables were GOSE, FIM, or CRS-R scores 6 months post-injury in patients with traumatic and hypoxic-ischemic etiologies, and the explanatory factors (regressors) all five miRNAs.

*p* values < 0.05 were considered significant. Statistical analyses were performed with GraphPad Prism 8.0.0 (GraphPad Software, San Diego, California, USA; http://www.graphpad.com).

## Results

### Participant characteristics

A total of 30 patients with prolonged DoC and 35 age-matched healthy controls were included in this study, as shown in Table 1 and Supplementary Table 2. Of the patient cohort, 20 were diagnosed with UWS and 10 with MCS. The underlying causes of DoC were TBI in 18 patients (15 of whom were involved in road accidents and 3 who suffered falls) and HIBI in 12 patients (10 of whom had cardiac arrests, 1 with opioid overdose, and 1 with asphyxia). There were no significant differences between TBI and HIBI patients in terms of age, sex, and time since brain injury (Table 1). However, the TBI group had more patients in MCS compared to the HIBI group. Additionally, patients with TBI had higher CRS-R and FIM scores at 6 months post-injury compared to those with HIBI (Table 1).

**Table 1:** Clinical and demographic characteristics of the study participants.

Characteristic	All patients (*n* = 30)	Healthy controls (*n* = 35)	*p*	TBI (*n* = 18)	HIBI (*n* = 12)	*p*
Males	28	34	0.5	17	11	0.8
Females	2	1		1	1	
Age (years)	34 (23–43)	39 (21–51)	0.7	29 (21–42)	36 (24–48)	0.2
Time since TBI (days)	41 (32–52)			36 (31–45)	48 (38.5–65)	0.1
UWS	20			9	11	0.02
MCS	10			9	1	
CRS-R score at admission	6 (4–8)			6.5 (4.3–11)	4.5 (3–6)	0.08
CRS-R score 6 months post-injury	7 (5–22)			22 (7–23)	5 (4–6.3)	< 0.001
GOSE score	2 (2–2.8)			2 (2–3.8)	2 (2–2)	0.06
FIM score	18 (18–49.3)			29 (18–78.8)	18 (18–18)	0.005

Data are reported as *n* or median (interquartile range). *CRS-R* Coma Recovery Scale-Revised, *EMCS* emergence from minimally conscious state, *FIM* Functional Independence Measure, *GOSE* extended Glasgow Outcome Scale, *HIBI* hypoxic-ischemic brain injury, *MCS* minimally conscious state, *TBI* traumatic brain injury, *UWS* unresponsive wakefulness syndrome.

### Changes in miRNA expression after TBI and HIBI

**Figure 1:**
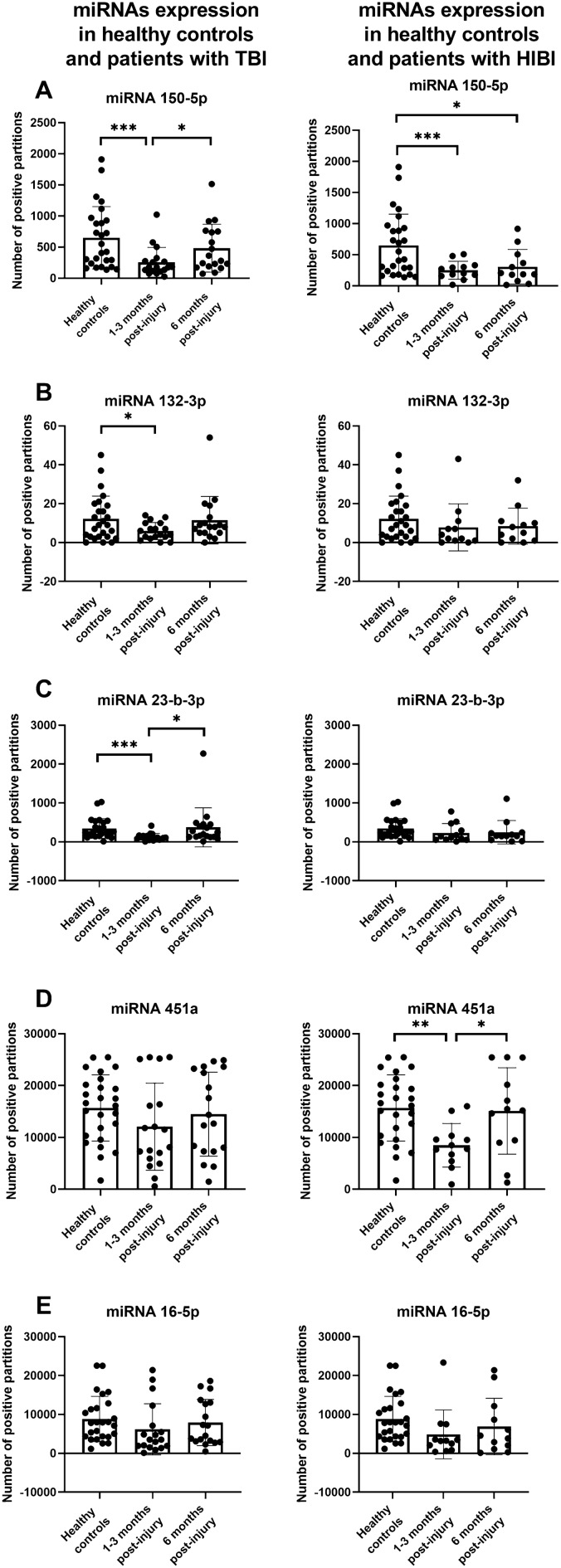
Comparison of serum miRNA levels between healthy controls and patients with DoC caused by traumatic brain injury (TBI) or hypoxic-ischemic brain injury (HIBI). The vertical lines represent the 95% confidence interval, the histograms indicate the median values, and the circles represent individual miRNA values.

At the time of study inclusion, miRNA 150-5p expression was reduced both in patients with TBI (*p* = 0.003) and HIBI (*p* = 0.01) compared to healthy controls (Fig. 1A). Six months post-injury, miRNA 150-5p expression returned to normal in patients with TBI, but not in patients with HIBI (*p* = 0.03). miRNAs 132-3p and 23-b-3p expressions were reduced only in patients with TBI at the time of study inclusion (*p* values = 0.04 and 0.001, respectively) (Fig. 1B,C). Conversely, miRNA 451a expression was reduced only in patients with HIBI at the time of study inclusion (*p* = 0.001) (Fig. 1D). miRNA 16-5p expression did not change compared to healthy controls (Fig. 1E). No significant differences were found in miRNA levels between TBI and HIBI patients at either time point.

### miRNAs dyshomeostasis after TBI and HIBI

**Figure 2:**
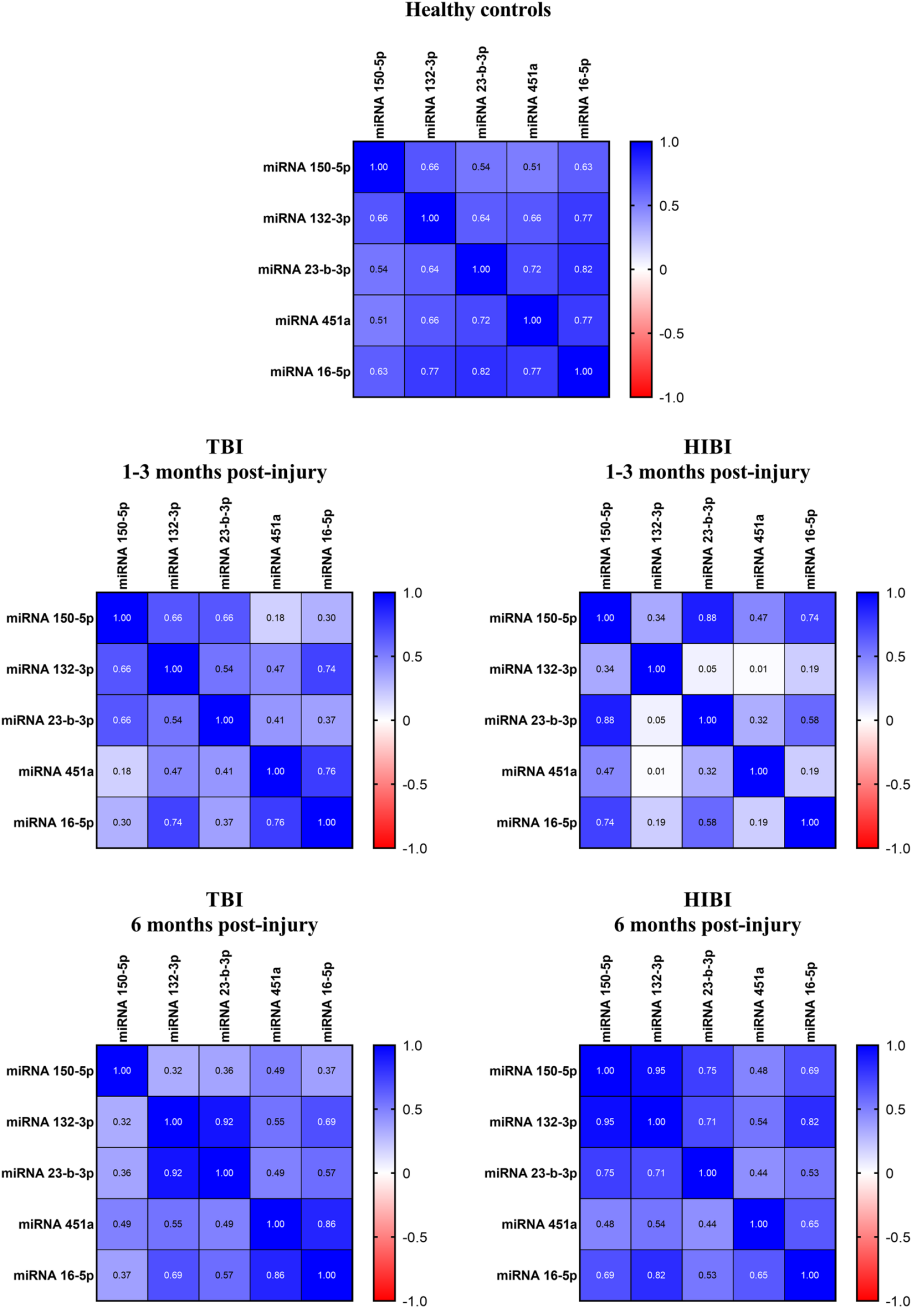
Pearson’s correlation matrix showing the relationship between the expression levels of the five miRNAs in healthy controls and patients with prolonged DoC caused by TBI or HIBI. The intensity of the color indicates the strength of the correlation coefficient. *HIBI* hypoxic-ischemic brain injury, *TBI* traumatic brain injury.

Pearson’s correlation matrixes showed that the expression of the selected miRNAs was highly interrelated in healthy subjects, but this correlation was generally lost after the brain injury (Fig. 2 and Supplementary Table 3). Six months post-injury, most combinations realigned with the normal trend, showing positive linear correlations (Fig. 2 and Supplementary Table 3). This trend was observed in both TBI and HIBI patients, but with a more evident dyshomeostasis in the latter at baseline. Despite some differences related to etiology and evaluation timing, this data indicates a significant disruption in the reciprocal expression of miRNAs 150-5p, 132-3p, 23b-3p and 451a, with only miRNA16-5p retaining a strong correlation with the other miRNAs both at baseline and 6 months post-injury.

### Correlation between miRNAs levels and clinical outcomes

**Figure 3:**
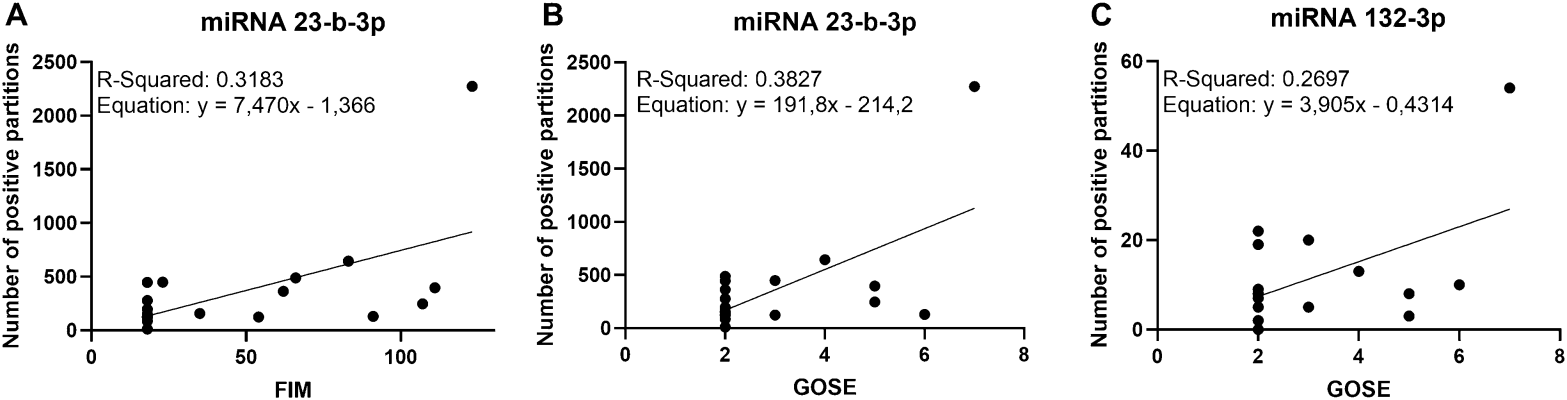
Linear correlations between FIM and GOSE scores and the number of positive partitions for miRNAs 23-b-3p and 132-3p, in patients with TBI. The graphs show the relationship between the FIM and GOSE scores and the number of positive partitions for miRNAs 23-b-3p and 132-3p, with the corresponding values of R^2^ and the equations obtained.

Multiple regression analysis showed a positive correlation between miRNA 23b-3p levels 1–3 months post-injury and both FIM (*p* = 0.01) and GOSE (*p* = 0.006) scores 6 months post-injury in patients with TBI (Fig. 3A,B). Additionally, a positive correlation was found between miRNA 132-3p levels and GOSE scores in patients with TBI (*p* = 0.03) (Fig. 3C). No other significant correlations were observed.

## Discussion

In this longitudinal study on patients with prolonged DoC, we found that a severe brain injury triggers a reorganization of miRNAs expression that (i) depends on the time elapsed since injury, (ii) is etiology-independent for some miRNA changes, and (iii) is etiology-specific for other miRNA alterations. In comparison to healthy controls, patients with TBI exhibited lower levels of miRNAs 150-5p, 132-3p, and 23-b-3p at baseline, while patients with HIBI had lower levels of miRNA 150-5p at baseline and 6 months post-injury and a reduction of miRNA 451a at baseline. Moreover, higher levels of miRNA 132-3p and miRNA 23b-3p were associated with better outcomes in patients with TBI, as demonstrated by improved GOSE and FIM scores 6 months post-injury.

Our findings concur with previous research indicating a reduction in miRNA 150-5p expression both in a mouse model of TBI^29^ and in patients with Alzheimer's and Parkinson's diseases^30,31^, suggesting that miRNA 150-5p is altered in a wide range of pathological conditions from acute brain injury to neurodegenerative diseases, making it a potential marker of brain injury independently of its etiology. Our study further showed that miRNA 150-5p expression was down-regulated in patients with DoC, both after TBI and HIBI. One potential explanation for the link between miRNA-150-5p dysregulation and various pathological conditions is its involvement in neuroinflammation. There is evidence of prolonged microglial activation after severe TBI and HIBI^32–34^ and miRNA-150-5p may inhibit the release of proinflammatory cytokines such as interleukine-1β, interleukine-6, and tumor necrosis factor-α by binding to the protein kinase AKT3^31^. Additionally, studies in patients with major trauma have shown that miRNA 150-5p targets the mRNA coding for protein kinase C alpha, a key player in inflammation^35^. Thus, the down-regulation of miRNA-150-5p may contribute to persistent neuroinflammation following brain injury, and increasing its expression may offer a new strategy for reducing neuroinflammation. In contrast to patients with TBI, downregulation of miRNA-150-5p persisted up to 6 months post-injury in patients with HIBI. This suggests that in these patients, whose outcomes were worse than those with TBI, the biochemical relevant pathways dependent on miRNA 150-5p remain dysregulated for an extended period post-injury.

The expression of miRNA 451a was reduced in patients with HIBI 1–3 months post-injury, making it a time- and etiology-specific marker of brain injury that may be related to inflammation. The down- or up-regulation of miRNA 451a has been shown to either increase or decrease neuroinflammation by modulating the levels of tumor necrosis factor α, interleukin‑1b, interleukin‑6, and interleukin‑18^36^.

In patients with TBI, miRNAs 132-3p and 23-b-3p were down-regulated 1–3 months after injury, making them specific markers for the timing and etiology of brain injury. The miRNA132 family is involved in the formation of neuronal connections and in synaptic plasticity. Post-mortem studies have revealed a decrease in miRNA 132-3p in the hippocampus, prefrontal cortex, and temporal cortex of patients with Alzheimer’s disease, including those in the early stages of disease^37,38^. miRNA 132 has been linked to various pathways, including astrocyte-related inflammation^39^, synaptic structure and plasticity^40,41^, and cholinergic signaling^42^, all of which play key roles in Alzheimer's disease. TBI increases the risk of developing dementia, including Alzheimer's disease, likely via mechanisms of long-term neurodegeneration^43,44^. Thus, the decrease in miRNA 132-3p levels may affect brain plasticity and contribute to neurodegeneration in patients with prolonged post-traumatic DOC, impacting their outcomes. After 6 months, miRNA 132-3p levels returned to normal values when most patients with post-traumatic DOC showed improvements in their level of consciousness. Therefore, the upregulation of miRNA 132-3p could have potential implications for brain plasticity and the recovery of consciousness in post-traumatic DoC, as demonstrated by its association with improved cognitive abilities in animal models^45^.

The expression of miRNA 23-b-3p exhibited a similar pattern to miRNA 132-3p, with a decrease in patients with TBI at baseline. miRNA 23-b-3p plays a role in regulating neuronal apoptosis and is involved in various cellular pathways in both physiological and pathological conditions^20^. A previous study on TBI patients and animal models has shown that miRNA 23-b levels decrease after TBI, and that overexpression of miRNA 23-b reduces neuronal autophagy in the hippocampus by targeting autophagy-related genes^46^. Our study expands upon this previous work by investigating patients with more severe injuries and a different temporal window (1–3 months post-injury compared to 24 h post-injury) and demonstrating that miRNA 23-b-3p levels recover to normal after 6 months. This finding is significant because it provides new insights into the dynamic changes in miRNA 23-b-3p levels after TBI and highlights the potential for miRNA 23-b-3p as a biomarker for post-traumatic DoC.

The levels of miRNA 16-5p, which have been linked to apoptosis induction and cell proliferation^47,48^, did not differ between healthy controls and patients with TBI or HIBI at 1–3 months and 6 months post-injury. However, a previous study reported reduced levels of miRNA 16-5p in plasma of patients with severe TBI 25–72 h after injury compared to healthy controls or patients with orthopedic injury^14^. Therefore, our findings suggest that the downregulation of miRNA 16-5p occurs only in the early phase after TBI.

In terms of clinical relevance, the most noteworthy finding was the correlation between the levels of miRNAs 132-3p and 23-b-3p 1–3 months post-injury and GOSE and FIM scores. Patients with higher expression of these miRNAs displayed better functional outcomes 6 months after injury. Previous studies have linked decreased levels of miRNAs 132-3p and 23-b-3p with neurodegeneration in both animal models and patients with Alzheimer's disease, while increased levels have been related to enhanced cognitive function^37,45,46,49^. Mechanisms of neurodegeneration and impaired plasticity have been described in patients with prolonged DoC, such as amyloid-β deposition^50^, gray and white matter atrophy^51–53^, and decreased expression of brain-derived neurotrophic factor^54^. Elevated levels of miRNAs 132-3p and 23-b-3p may counteract neurodegeneration and promote brain plasticity in patients with DoC, leading to better outcomes. So, miRNAs 132-3p and 23-b-3p are promising candidates for inclusion in prognostic models for patients with prolonged DoC and warrant further investigation as potential targets for therapeutic intervention.

Studying the overall changes in miRNA expression after a brain injury can provide a more comprehensive view of the impact of severe brain injuries on the miRNA regulatory network. We observed a loss of reciprocal correlations among miRNAs, which was more pronounced 1–3 months post-injury and in patients with HIBI. The loss of reciprocal correlations among miRNAs may reflect a disruption of the complex molecular network regulating neuronal survival and plasticity, and this dysregulation is more profound in patients with HIBI. Furthermore, the more pronounced dysregulation in the acute phase after injury suggests that this is a critical time window for therapeutic intervention. The complex and simultaneous regulation of multiple biochemical pathways by each miRNA makes it challenging to predict the final effects on neuronal survival and plasticity. Our understanding of this field is still limited, and further research is needed, such as developing computational models to predict the combined effect of multiple miRNAs on patient outcomes.

Two recent studies have investigated changes in miRNA expression in patients with prolonged DoC^55,56^. Although they focused on different miRNAs or employed different methodologies compared to our study, their findings are consistent in suggesting that miRNAs represent an intriguing target for evaluation in prolonged DoC. In patients with TBI, HIBI, or subarachnoid hemorrhage, Petrova and colleagues identified significant differences in the expression of miRNAs 93-5p, 21-5p, and 191-5p in both cerebrospinal fluid and plasma, depending on the specific etiologies^55^. Zilliox and colleagues examined the whole blood miRNA profile in six patients with post-traumatic DoC lasting an average of 1.5 years, discovering 48 miRNAs that were differentially expressed compared to controls^56^.

While the findings of this study provide valuable insights into the potential role of miRNAs as biomarkers for prognosis and targets for therapeutic intervention in patients with prolonged DoC, there are several limitations to consider. One of the primary limitations is the small sample size, especially concerning HIBI and female patients, which may limit the generalizability of the results. Moreover, we evaluated miRNAs in the blood only, while analyzing miRNAs in the cerebrospinal fluid may possibly provide more accurate results regarding the changes occurring in the central nervous system^55,57^. Additionally, our assessment of outcomes was limited to a follow-up period of 6 months post-injury. While this time frame may be adequate to capture most of the recovery in patients with non-TBI etiologies, it may not fully evaluate the complete recovery of patients with TBI, who may continue to improve up to 1-year post-injury^3^. Finally, while the study focused on miRNA dysregulation, it must be added that miRNAs are just one of the many potential molecular mechanisms dysregulated in DoC, and further research is needed to fully understand the particularly complex pathophysiology of this condition.

In conclusion, this study offers important insights into the potential of miRNAs for understanding the molecular mechanisms of DoC as well as for enhancing prognosis and treatment in patients with prolonged DoC. However, the limited sample size and the need for further research to fully understand the complex interactions of miRNAs and their effects on neuronal survival and plasticity should be considered. Further studies with larger sample sizes and longer follow-up periods could help validate and expand upon these findings.

### Supplementary Information

Supplementary Tables.

## Data Availability

The data supporting the findings of the study are available upon request from the corresponding author (S.B.).
