# Supplementary material for: Investigating microRNAs as biomarkers in disorders of consciousness: a longitudinal multicenter study
Source: Sci Rep. 2023 Oct 27;13:18415. doi: 10.1038/s41598-023-45719-7 (PMC10611795; doi:10.1038/s41598-023-45719-7)
Supplement: Supplementary file 1 — Supplementary Tables. [file 41598_2023_45719_MOESM1_ESM.docx]

| **Product Name** | **miRBase ID** | **miRBase Accession** | **Catalog Number** |
| --- | --- | --- | --- |
| miRCURY LNA miRNA PCR Assay (cat.no 339306, QIAGEN) | hsa-miR-16-5p | MIMAT0000069 | YP00205702 |
|  | hsa-miR-150-5p | MIMAT0000451 | YP00204660 |
|  | hsa-miR-132-3p | MIMAT0000426 | YP00206035 |
|  | hsa-miR-23b-3p | MIMAT0000418 | YP02119314 |
|  | hsa-miR-451a | MIMAT0001631 | YP02119305 |

**Supplementary Table 1.** Locked nucleic acid primers used for detecting miRNA targets.

**Supplementary Table 2**. Patients’ demographic and clinical details.

| **Pt** | **Sex** | **Age (y)** | **Brain injury** | **Time since**  **brain injury (days)** | **DoC** | **CRS-R score at admission** | **CRS-R score 6 months post-injury** | **GOSE** | **FIM** | **Main CT and / or MRI findings*** |
| --- | --- | --- | --- | --- | --- | --- | --- | --- | --- | --- |
| 1 | M | 37 | HIBI | 65 | UWS | 3 | 5 | 2 | 18 | Bi-hemispheric hypodensity |
| 2 | M | 65.4 | HIBI | 46 | UWS | 2 | 3 | 2 | 18 | None |
| 3 | M | 56.4 | TBI | 46 | MCS | 7 | 11 | 2 | 18 | Left hemispheric subdural hematoma |
| 4 | M | 19 | TBI | 30 | UWS | 5 | 22 | 3 | 23 | Diffuse axonal injury; cerebral edema |
| 5 | M | 42.8 | TBI | 33 | MCS | 18 | 23 | 4 | 83 | Diffuse axonal injury |
| 6 | M | 24.3 | HIBI | 64 | UWS | 6 | 7 | 2 | 18 | None |
| 7 | F | 34.8 | HIBI | 37 | UWS | 4 | 5 | 2 | 18 | None |
| 8 | M | 26 | TBI | 43 | MCS | 15 | 23 | 3 | 54 | Right fronto-temporal cortical contusions |
| 9 | M | 20.4 | TBI | 90 | UWS | 4 | 4 | 2 | 18 | Right frontal epidural hematoma; multiple cortical contusions; cerebral edema |
| 10 | F | 30.4 | TBI | 47 | UWS | 6 | 7 | 2 | 18 | Diffuse axonal injury |
| 11 | M | 39.7 | TBI | 38 | MCS | 8 | 10 | 2 | 18 | Bilateral frontal cortical contusions; cerebral edema |
| 12 | M | 22.9 | TBI | 30 | MCS | 8 | 22 | 2 | 35 | Right temporo-parietal epidural hematoma |
| 13 | M | 22.5 | TBI | 30 | UWS | 3 | 5 | 2 | 18 | Left hemispheric epidural hematoma; ischemic lesions in the brainstem |
| 14 | M | 27.7 | TBI | 33 | MCS | 14 | 23 | 2 | 62 | Diffuse axonal injury |
| 15 | M | 17.8 | TBI | 33 | MCS | 12 | 23 | 2 | 66 | Left lenticular nucleus hemorrhage |
| 16 | M | 51.1 | TBI | 42 | UWS | 2 | 23 | 5 | 111 | Diffuse axonal injury |
| 17 | M | 43.7 | HIBI | 30 | UWS | 5 | 6 | 2 | 18 | Bilateral hypoxic lesions in the occipital cortex, thalamus, and hippocampus |
| 18 | M | 34.1 | HIBI | 65 | UWS | 2 | 5 | 2 | 18 | Cerebral edema |
| 19 | M | 18.2 | TBI | 52 | MCS | 18 | 23 | 5 | 107 | Bilateral frontal subdural hematoma |
| 20 | M | 24.2 | HIBI | 40 | UWS | 3 | 2 | 2 | 18 | Bi-hemispheric hypodensity; cerebral edema |
| 21 | M | 58.3 | TBI | 30 | UWS | 6 | 23 | 6 | 91 | Left hemispheric epidural hematoma; right hemispheric subdural hematoma |
| 22 | M | 42.4 | TBI | 32 | UWS | 4 | 7 | 2 | 18 | Left lenticular nucleus hemorrhage; multiple cortical contusions |
| 23 | M | 18.1 | TBI | 42 | MCS | 6 | 23 | 7 | 123 | Diffuse axonal injury |
| 24 | M | 48.2 | HIBI | 30 | UWS | 5 | 4 | 2 | 18 | Cerebral edema |
| 25 | M | 33.3 | TBI | 30 | UWS | 2 | 3 | 2 | 18 | Diffuse axonal injury |
| 26 | M | 24.2 | HIBI | 39 | UWS | 6 | 6 | 2 | 18 | Cerebral edema |
| 27 | M | 19.1 | HIBI | 50 | UWS | 4 | 4 | 2 | 18 | Cerebral edema |
| 28 | M | 38.4 | TBI | 90 | UWS | 7 | 7 | 2 | 18 | Multiple cerebral and cerebellar contusions |
| 29 | M | 48.6 | HIBI | 87 | UWS | 8 | 7 | 2 | 18 | Cerebral edema |
| 30 | M | 51.4 | HIBI | 90 | MCS | 10 | 11 | 3 | 20 | Cerebral edema |

*When available, the data are from immediately after brain injury; otherwise, the first data available is shown.

CRS-R, Coma Recovery Scale–Revised; CT, computed tomography; DoC, disorder of consciousness; F, female; FIM, Functional Independence Measure; GOSE, Glasgow Outcome Scale Expanded; HIBI, hypoxic-ischemic brain injury; M, male; MCS, minimally conscious state; MRI, magnetic resonance imaging; Pt, patient; TBI, traumatic brain injury; UWS, unresponsive wakefulness syndrome.

**Supplementary Table 3.** p-values for miRNA expression in healthy controls, patients with TBI, and patients with HIBI.

| **HEALTHY CONTROL** | | | | |
| --- | --- | --- | --- | --- |
|  | *miRNA 16-5p* |  |  |  |
| *miRNA 150-5p* | < 0.001 | *miRNA 150-5p* |  |  |
| *miRNA 132-3p* | < 0.001 | < 0.001 | *miRNA 132-3p* |  |
| *miRNA 23b-3p* | < 0.001 | 0.004 | < 0.001 | *miRNA 23b-3p* |
| *miRNA 451a* | < 0.001 | 0.008 | < 0.001 | < 0.001 |
|  |  |  |  |  |
| **TBI 1-3 months post-injury** | | | | |
|  | *miRNA 16-5p* |  |  |  |
| *miRNA 150-5p* | 0.2 ↓ | *miRNA 150-5p* |  |  |
| *miRNA 132-3p* | < 0.001 ↓ | 0.002 ↓ | *miRNA 132-3p* |  |
| *miRNA 23b-3p* | 0.1 ↓ | 0.002 ≈ | 0.02 ↓ | *miRNA 23b-3p* |
| *miRNA451a* | < 0.001 ↓ | 0.4 ↓ | 0.05 ↓ | 0.09 ↓ |
|  |  |  |  |  |
| ***6 months post-injury*** | | | | |
|  | *miRNA 16-5p* |  |  |  |
| *miRNA 150-5p* | 0.1 ↓ | *miRNA 150-5p* |  |  |
| *miRNA 132-3p* | 0.001 ≈ | 0.2 ↓ | *miRNA 132-3p* |  |
| *miRNA 23b-3p* | 0.01 ↓ | 0.1 ↓ | < 0.001 ↑ | *miRNA 23b-3p* |
| *miRNA451a* | < 0.001 ≈ | 0.03 ≈ | 0.02 ≈ | 0.04 ↓ |

| **HIBI 1-3 months post-injury** | | | | |
| --- | --- | --- | --- | --- |
|  | *miRNA 16-5p* |  |  |  |
| *miRNA 150-5p* | 0.005 ≈ | *miRNA 150-5p* |  |  |
| *miRNA 132-3p* | 0.5 ↓ | 0.2 ↓ | *miRNA 132-3p* |  |
| *miRNA 23b-3p* | 0.04 ↓ | < 0.001 ↑ | 0.8 ↓ | *miRNA 23b-3p* |
| *miRNA451a* | 0.5 ↓ | 0.1 ↓ | 0.9 ↓ | 0.3 ↓ |
| **6 months post injury** | | | | |
|  | *miRNA 16-5p* |  |  |  |
| *miRNA 150-5p* | 0.01 ≈ | *miRNA 150-5p* |  |  |
| *miRNA 132-3p* | <0.001 ≈ | <0.001 ↑ | *miRNA 132-3p* |  |
| *miRNA 23b-3p* | 0.07 ↓ | 0.004 = | 0.01 ≈ | *miRNA 23b-3p* |
| *miRNA451a* | 0.02 ≈ | 0.1 ↓ | 0.07 ↓ | 0.1 ↓ |

Arrows indicate significance compared to controls: ↓ = lower/absent significance, ↑ = higher significance, and ≈ = no significant difference. HIBI: hypoxic-ischemic brain injury; TBI: traumatic brain injury.
